# Exploratory Clinical Trial to Evaluate the Efficacy of a Topical Traditional Chinese Herbal Medicine in Psoriasis Vulgaris

**DOI:** 10.1155/2015/719641

**Published:** 2015-03-05

**Authors:** Yuhe Yan, Wali Liu, Philippe Andres, Colette Pernin, Laurent Chantalat, Philippe Briantais, Albert Lin, Lilian Feng

**Affiliations:** ^1^Department of Dermatology, Guang'anmen Hospital, China Academy of Chinese Medical Sciences, 100053 Beijing, China; ^2^Galderma R&D, Les Templiers, 2400 route des Colles, BP 87, 06902 Sophia Antipolis Cedex, France

## Abstract

*Objective*. To evaluate the efficacy and safety of herbal ointment, Shi Du Ruan Gao, in patients with plaque-type psoriasis. *Design*. Single-center, randomized, investigator-blinded, parallel group, placebo-controlled study. *Participants*. One hundred outpatients with mild to moderate chronic plaque-type psoriasis were enrolled. *Intervention*. The patients applied either Shi Du Ruan Gao ointment or vehicle ointment topically to for 8 weeks. *Main Outcome Measures*. The outcomes were assessed using the following criteria: Total Severity Score (TSS, sum of erythema, scaling, and plaque elevation/induration, on a 0 to 4 scale), Investigator Global Assessment (IGA) evaluated on a 0 (Clear) to 4 (s to very severe) scale, and Global Subjects' Assessment of treatment response on a 7-point scale from −1 (worse) to 5 (Cleared). *Results*. Significant reductions in the Total Severity Score (*P* < 0.001) (mean score: 2.7 after Shi Du Ruan Gao treatment versus 5.1 in control subjects). Both Investigator Global Assessment (IGA) and Global Subjects' Assessment of treatment are better in the Shi Du Ruan Gao group than the control group (*P* < 0.001). *Conclusion*. Shi Du Ruan Gao ointment was a safe, and effective therapy for plaque-type psoriasis.

## 1. Introduction

Psoriasis is a common, chronic, relapsing, immune-mediated skin disease. The skin lesions seen in psoriasis may include red, scaly patches, papules, and plaques, which varies in severity from minor localized patches to complete body coverage. Psoriasis affects 2–4% of the general population worldwide. There are five types of psoriasis including plaque, gluttate, inverse, pustular, and erythrodermic psoriasis. However, chronic plaque psoriasis is the most common form of psoriasis, which is characterized by hyperkeratosis (thickening of the horny layer of the skin), induration, scale, and erythema. Psoriasis could be considered as a relatively mild disease but could have profound physical and psychosocial effects on those affected patients. Although the causes of psoriasis are not completely understood, psoriasis develops when the immune system mistakes the normal skin cells as pathogen and triggers inflammatory reaction. The triggers for psoriasis have been suggested as oxidative stress, stress, and withdrawal of a systemic corticosteroid.

In Chinese medicine, this disease is called “Bai Bi,” or “Song Pi Xian.” Professor Renkang Zhu (1908–2000) was one of the most famous traditional Chinese medicine (TCM) dermatologists. He studied psoriasis for over 70 years. During those years, he established many herbal decoctions and ointments for treating psoriasis. Many of them had good response in clinic. According to Professor Zhu, psoriasis can be referred to as “domination of evil heat in the blood.” The evil heat in the blood could be due to invasion of six pathogens and internal injury from seven emotions according to TCM principle. Psoriasis could develop from the excess of blood evil heat stagnating in the muscles and skin to form eruptions. In addition, excess of blood evil heat can induce wind and excessive wind can cause dryness. Both of wind and dryness could result in pruritus and scales. Professor Zhu called this condition as “blood heat and wind dryness.” Therefore, TCM principle of treating psoriasis was developed more than twenty years ago as “clearing heat and cooling blood,” which is represented as the herbal ointment, Shi Du Ruan Gao (SDRG). SDRG was established in 1955, which was used in Guang'anmen Hospital for treating psoriasis for almost 60 years.

## 2. Methods

### 2.1. Study Design

This is a single-center, randomized, investigator-blinded, parallel group, placebo-controlled clinical investigation to demonstrate the efficacy of a herbal ointment on patients suffering from mild to moderate chronic plaque psoriasis over a period of 8 weeks. The protocol was approved by the Institutional Review Boards for Human Studies of Guang'anmen Hospital.

**Figure 1:**
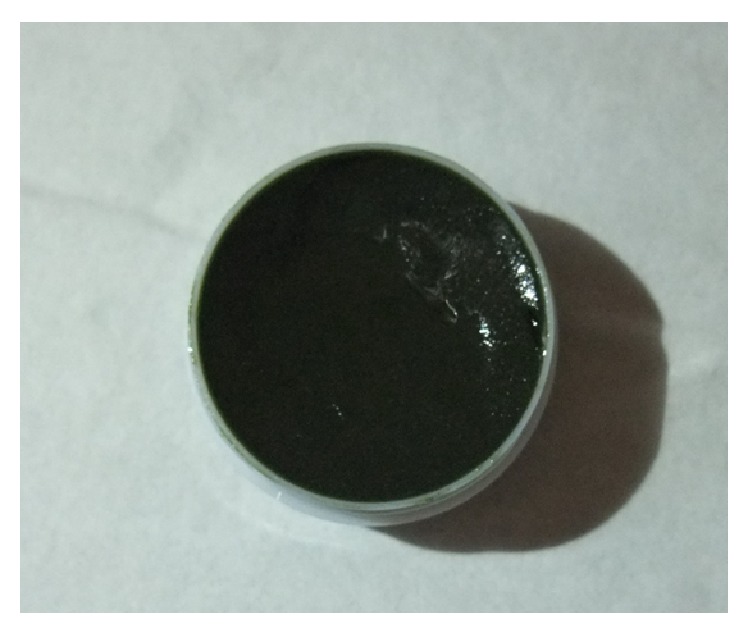
SHI DU RUAN GAO contains indigo naturalis (Qing Dai), Cortex Phellodendri (Huang Bai), Gypsum fibrosum preparatum (Duan Shi Gao), Calamine (Lu Gan Shi), and Galla chinensis (Wu Bei Zi).

*Participants*. From November 2009 to October 2010, 149 psoriasis patients have been screened. One hundred patients with stable mild to moderate chronic plaque-type psoriasis met the inclusion criteria and were enrolled in the study. They were randomly assigned to treatment (SDRG) and placebo groups, respectively as indicated in Figure 1. The inclusion criteria are (1) age from 18 to 65 years old and (2) stable mild to moderate chronic plaque-type psoriasis covering between 5 and 20% of body surface area (BSA). Exclusion criteria include (1) rapidly progressing unstable psoriasis; (2) other types of psoriasis such as acute gluttate, inverse, pustular, erythrodermic, and arthropathic psoriasis; (3) patients used topical antipsoriasis medications (including phototherapy) within the past 2 weeks; (4) patients used systemic antipsoriasis medications within the past 8 weeks; (5) persons foresee pregnancy and/or intensive UV exposure during the study such as sunbathing and tanning; (6) persons cannot communicate or cooperate with the investigator due to language and mental issues.

### 2.2. Intervention

SDRG consists of five TCM herbs including indigo naturalis (Qing Dai), Cortex Phellodendri (Huang Bai), Gypsum (Duan Shi Gao), Smithsonite (Lu Gan Shi), and Gallae Rhois Chinensis (Wu Bei Zi). The ointment was produced by Guang'anmen Hospital, China Academy of Chinese Medical Sciences (Website: http://www.gamhospital.ac.cn/) with rigorous control. The placebo was made of silicone oil ointment (dimeticone). The ointment of SDRG was shown in Figure 1. The SDRG and dimeticone were applied to plaque psoriasis twice a day (after getting up and before going to bed) to cover the area completely for 8 weeks.

## 3. Outcome Measure

Patients were scheduled to have an initial visit and then follow-up visits at 4 and 8 weeks. At each visit, psoriasis areas were visualized and measured. General condition of patients such as itching was evaluated. The efficacy assessments consisted of Total Severity Score (TSS, sum of erythema, scaling, and plaque elevation, on a 0 to 4 scale), each individual score, Investigator Global Assessment (IGA), which evaluated investigators' judgments for the improvement of lesions during and after the treatment on a scale of 0 (clear) to 4 (very severe), and the Global Subjects' Assessment of Improvement (GSAI), which evaluated subjects' judgments for the improvement of lesions during and after the treatment on a 7-point scale from −1 (worse) to 5 (cleared). Adverse events of skin area such as local erythema were evaluated.

### 3.1. Statistical Analyses

Statistical analyses were performed by SAS version 8.2.

The TSS at week 8 (ITT-LOCF) and also overtime was submitted to an analysis of covariance including treatment group as factor and the TSS at baseline as a covariate.

Due to skewed distributions, change from baseline in individual scores, change from baseline in Investigator Global Assessment score, and Global assessment of improvement by the subjects were submitted to a Cochran Mantel Haenszel test.

Success rate (defined as 0 = clear or 1 = very mild) for IGA was submitted to a Chi-square test, except if a theoretical frequency in a cell was below 5; the Fisher's exact test was to be used.

For all efficacy variables in the ITT analyses, the last observation carried forward (LOCF) imputation method was applied to handle the dropouts or missing data.

All tests were two-sided and significance was declared at the 5% level.

## 4. Results

### 4.1. Participant Enrollment and Baseline of Characteristics

Total of 100 patients from a single-center were randomized into either SDRG (*N* = 50) or dimeticone/placebo (*N* = 50) groups as the intent-to-treat (shown in Table 1). However, there were seven patients withdrawn from the study: five from SDRG group and 2 from placebo group.

The baseline characteristics of all patients were listed in Table 1. All patients had a BSA of at least 5%. Of the 100 subjects, 64 were males (34 in SDRG group and 30 in dimeticone group) and 36 were females (16 in SDRG group and 20 in dimeticone group). Age ranged between 21 and 64 years with a mean around 45.8 years (45 in the dimeticone group and 47 in the SDRG group resp.). The mean of TSS was 6.4 ± 1.6 in SDRG group and was 6.5 ± 1.3 in dimeticone group.

### 4.2. Primary Outcomes

**Figure 2:**
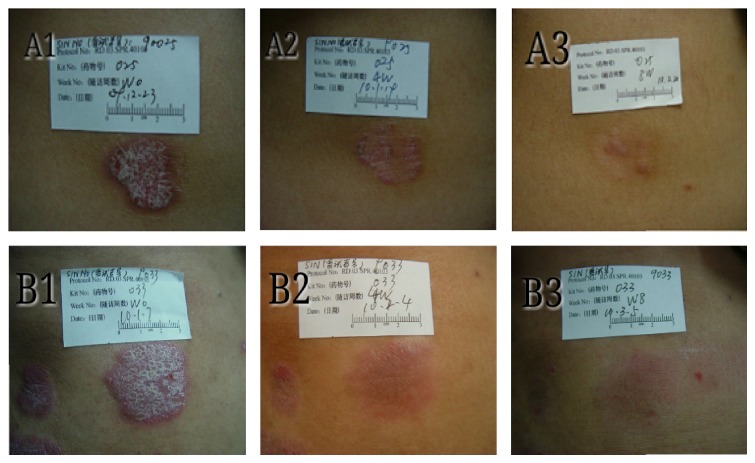
Clinical photographs of 2 patients with plaque-type psoriasis before, after 4 weeks, and after 8 weeks of treatment with SDRG.

Morphologies of the psoriasis plaque at the beginning of treatment and at 4 and 8 weeks after treatment from each patient of each group were shown in Figure 2. Plaque elevation and scaling were significantly reduced after SDRG treatment as compared to that of dimeticone treatment. The TSS improvements were shown in Table 2. The mean of TSS changed from 6.4 ± 1.6 to 5.1 ± 2.1 in dimeticone group and from 6.5 ± 1.3 to 2.7 ± 2.2 in SDRG group. The outcomes got significant difference in each group and between the two groups (*P* < 0.001).

### 4.3. Secondary Outcomes

The secondary outcomes of the Investigator Global Assessment (IGA) and Subject's Global Assessment of Improvement (GSAI) were shown in Tables 3, 4, and 5, respectively. There were 72% success rate in SDRG group and only 24% in dimeticone groups. There was a statistical significance between the two groups. At the last visit of patients, GSAI was rated from worse to clear. Most patients in the SDRG group indicated fair (24%), good (24%), excellent (33%), and clear (7%) while most patients in dimeticone group indicated unchanged (29%), slight change (27%), and fair (29%).

## 5. Adverse Events

No severe adverse events were observed during the treatment such as erythema. Only two mild worsening of psoriasis was reported in the dimeticone group and there were no cases in the SDRG group (shown in Table 6).

## 6. Discussion

This study was to assess the clinical benefit and safety of SDRG in patients with plaque psoriasis and to provide evidence-based research to demonstrate the TCM therapy for psoriasis. The results clearly exhibited that SDRG treatment significantly improved the TSS, IGA, and GSAI compared to those in placebo groups. The composition of SDRG is according to the treatment principle of Dr. Zhu in our hospital, clearing heat and cooling blood. The five herbs in the ointment have the function to clear heat and cool blood.

Indigo naturalis is a dark blue powder prepared from the leave of* Indigofera tinctoria*. According to the TCM theory, indigo naturalis could remove heat and dissolve heat toxins. Recent research revealed that indigo naturalis is useful herb for following purposes such as antipyretic, anti-inflammation, antivirus, antimicrobial, anticancer, and detoxification [1, 2]. Several clinical studies showed that indigo naturalis and one of its active ingredients, indirubin, were effective in treating psoriasis [3–6].

Cortex Phellodendri is derived from the dried bark of* Phellodendron chinense* and are used in clinical practice for centuries in Chinese medicine for the treatment of various inflammatory conditions. It could clear away heat, dry up dampness, and stop itching. It contains several chemicals such as berberine, palmatine, jatrorrhizine, phellodendrine, and magnoflorine. Recent experimental researches demonstrated that it could significantly downregulate lipopolysaccharide-induced interleukin-6 (IL-6), IL-1*β*, and macrophage chemoattractant protein-1 (MCP-1) in mice serum. In addition, Cortex Phellodendri inhibited inducible nitric oxide synthase (iNOS), activated nuclear factor-*κ*B (NF-*κ*B) by degradation and phosphorylation of I*κ*B*α*, and attenuated phosphorylation of mitogen-activated protein kinases such as ERK1/2, p38, and JNK in mice treated with lipopolysaccharide [7].

Gypsum is fried hydrous calcium sulphate. It has astringent and granulating functions according to TCM theory. Modern researches show it can accelerate the formation of collagenoblast and micrangium in wounds and the proliferation of granulation tissues, thus promoting the skin wounds to healing [8].

Calamine gets astringent and granulating functions in TCM theory. It was benefit for the reduction of wound area. In high dose promoted the formation of neoformative capillaries, which could increase the blood supply of the wound and promote its recovery [9].

Gallae Rhois Chinensis is Melaphis chinensis (Bell) Baker on Rhus chinensis Mill, Rhus potaninii Maxim, or Rhus punjabensis Stew.var.Sinica(Diels)Rehd.et Wils, which has “dissolving heat-toxin, astringing liquid and blood” functions in TCM theory. Laboratory researches show that it mainly contains tannic acid and has antibacterial effect [10].

With all these components, SDRG could clear away heat and cool the blood to promote granulation and heal the wound in TCM theory. Our study confirmed that SDRG has antipsoriasis effect. However, what is the mechanism of SDRG in the treatment of psoriasis according to Western medicine still remains unclear. It could be related to its regulation of immune systems and/or microcirculation. It could also be related to its antiproliferation and/or anti-inflammatory activities. Nevertheless, our clinical findings indicate that a topical herbal formula (SDRG) according to TCM principle could be used for improvement and treatment of patients with plaque-type psoriasis.

## Figures and Tables

**Table 1:** The baseline characteristics of all patients.

		Dimeticone/placebo	Shi Du Ruan Gao
Gender	*N* (%)	50	50
	Female	20 (40)	16 (32)
	Male	30 (60)	34 (68)

Age (in years)	*N* (%)	50	50
	<65 years	50 (100.0)	50 (100.0)
	Mean ± SD	44.7 ± 12.2	46.9 ± 11.3
	Median	45	50
	Min~Max	22~64	21~64

TSS (baseline)	Mean ± SD	6.4 ± 1.6	6.5 ± 1.3
	Median	6.0	6.0
	Min~Max	3~10	4~10
	*Q*1~*Q*3	5~8	4~10

**Table 2:** Summary of the TSS at baseline and week 8. ^(1)^Based on an ANCOVA with including the treatment group as factor and TSS at baseline as a covariate.

		Dimeticone/placebo	Shi Du Ruan Gao	*P* value^(1)^
Baseline	*N*	50	50	
	Mean ± SD	6.4 ± 1.6	6.5 ± 1.3	
	Median	6.0	6.0	
	Min~Max	3~10	4~10	
	*Q*1~*Q*3	5~8	4~10	

Week 8 LOCF (ITT)	*N*	50	50	<0.001
	Mean ± SD	5.1 ± 2.1	2.7 ± 2.2	
	LS-Mean	5.1	2.7	
	Median	5.0	2.0	
	Min~Max	0~10	0~8	
	*Q*1~*Q*3	4~6	1~4	

Week 8 (PP)	*N*	48	45	<0.001
	Mean ± SD	5.0 ± 2.1	2.4 ± 2.0	
	LS-Mean	5.1	2.3	
	Median	5.0	2.0	
	Min~Max	0~10	0~8	
	*Q*1~*Q*3	4~6	1~3	
	*Q*1~*Q*3	4~6	1~3	

**Table 3:** Summary of the secondary efficacy criteria (ITT-LOCF). ^(1)^CMH test using row mean score difference statistic and ridit transformation.

		Dimeticone/placebo		Shi Du Ruan Gao		*P* value^(1)^
		Raw data	Change from baseline	Raw data	Change from baseline	Change from baseline
TSS	*N*	50	50	50	50	<0.001
	Mean ± SD	5.1 ± 2.1	−1.3 ± 1.4	2.7 ± 2.2	−3.8 ± 1.9	
	Median	5.0	−1.0	2.0	−4.5	
	Min~Max	0~10	−5~1	0~8	−7~0	
	*Q*1~*Q*3	4~6	−2~0	1~4	−5~−3	

Erythema	*N*	50	50	50	50	<0.001
	Mean ± SD	1.9 ± 0.8	−0.3 ± 0.7	1.2 ± 0.7	−1.2 ± 0.7	
	Median	2.0	0.0	1.0	−1.0	
	Min~Max	0~4	−2~1	0~3	−2~1	
	*Q*1~*Q*3	1.0~2.0	−1.0~0.0	1.0~2.0	−2.0~1.0	

Plaque elevation	*N*	50	50	50	50	<0.001
	Mean ± SD	1.7 ± 0.8	−0.4 ± 0.6	0.8 ± 0.9	−1.5 ± 0.9	
	Median	2.0	0.0	0.5	−2.0	
	Min~Max	0~3	−2~1	0~3	−3~0	
	*Q*1~*Q*3	1.0~2.0	−1.0~0.0	0.0~1.0	−2.0~1.0	

Scaling	*N*	50	50	50	50	<0.001
	Mean ± SD	1.5 ± 0.9	−0.6 ± 0.8	0.8 ± 0.9	−1.1 ± 0.8	
	Median	1.0	0.0	1.0	−1.0	
	Min~Max	0~4	−2~1	0~3	−3~0	
	*Q*1~*Q*3	1.0~2.0	−1.0~0.0	0.0~1.0	−2.0~1.0	

**Table 4:** Distribution of IGA in terms of success rate at week 8 (ITT-LOCF). Success is defined as subjects having an IGA of 0 = clear or 1 = very mild ^(1)^Chi-square.

		Dimeticone/placebo	Shi Du Ruan Gao	*P* value^(1)^
IGA at week 8, LOCF	*N* (%)	50 (100)	50 (100)	<0.001
	Success	12 (24.0)	36 (72.0)	
	Failure	38 (76.0)	14 (28.0)	

**Table 5:** Subjects' Global Assessment of Improvement (observed cases). ^(1)^CMH test using row mean score difference statistic and ridit transformation.

		Dimeticone/placebo	Shi Du Ruan Gao	*P* value^(1)^
Final visit (observed cases)	*N* (%)	48 (100)	45 (100)	<0.001
	−1: worse	1 (2.1)		
	0: unchanged	14 (29.2)	2 (4.4)	
	1: slight	13 (27.1)	3 (6.7)	
	2: fair	14 (29.2)	11 (24.4)	
	3: good	4 (8.3)	11 (24.4)	
	4: excellent	2 (4.2)	15 (33.3)	
	5: cleared		3 (6.7)	

**Table 6:** Adverse events are defined as events occurred after the first use of medication. Numbers in columns cannot be added because a given subject may have reported more than one AE.

MedDRA v 12.0	Dimeticone/placebo (*N* = 50)		Shi Du Ruan Gao (*N* = 50)	
	*N* events	*N* (%) subjects	*N* events	*N* (%) subjects
All AEs	2	2 (4.0%)	0	0 (0.0%)
Related AEs	0	0 (0.0%)	0	0 (0.0%)
All dermatologic AEs	2	2 (4.0%)	0	0 (0.0%)
Related dermatologic AEs	0	0 (0.0%)	0	0 (0.0%)
All serious AES	0	0 (0.0%)	0	0 (0.0%)
Related serious AEs	0	0 (0.0%)		0 (0.0%)
Severe AEs	0	0 (0.0%)	0	0 (0.0%)
Related severe AEs	0	0 (0.0%)	0	0 (0.0%)
AEs of special interest	0	0 (0.0%)	0	0 (0.0%)
Related AEs of special interest	0	0 (0.0%)	0	0 (0.0%)
AEs leading to discontinuation	0	0 (0.0%)	0	0 (0.0%)
Related AEs leading to discontinuation	0	0 (0.0%)	0	0 (0.0%)
Deaths	0	0 (0.0%)	0	0 (0.0%)
